# α-Solanine Inhibits Proliferation, Invasion, and Migration, and Induces Apoptosis in Human Choriocarcinoma JEG-3 Cells In Vitro and In Vivo

**DOI:** 10.3390/toxins13030210

**Published:** 2021-03-13

**Authors:** Ting Gu, Wei Yuan, Chen Li, Zhilong Chen, Yuting Wen, Qiyi Zheng, Qing Yang, Xingyao Xiong, Anwen Yuan

**Affiliations:** 1 College of Veterinary Medicine, Hunan Agricultural University, Changsha 410128, China; gut9592@stu.hunau.edu.cn (T.G.); WeiYuan1997@stu.hunau.edu.cn (W.Y.); lichen.0904@foxmail.com (C.L.); zlongchen@stu.hunau.edu.cn (Z.C.); yuting@stu.hunau.edu.cn (Y.W.); qyzheng@stu.hunau.edu.cn (Q.Z.); 2 Shenzhen Agricultural Genome Research Institute, Chinese Academy of Agricultural Sciences, Shenzhen 518120, China

**Keywords:** α-Solanine, choriocarcinoma cells, proliferation, migration, invasion, apoptosis

## Abstract

α-Solanine, a bioactive compound mainly found in potato, exhibits anti-cancer activity towards multiple cancer cells. However, its effects on human choriocarcinoma have not been evaluated. In the present study, we investigated the effect of α-solanine on cell proliferation and apoptosis in human choriocarcinoma in vitro and in vivo. The results showed that α-solanine, at concentrations of 30 μM or below, did not affect the cell viability of the choriocarcinoma cell line JEG-3. However, colony formation was significantly decreased and cell apoptosis was increased in response to 30 μM α-solanine. In addition, α-solanine (30 μM) reduced the migration and invasion abilities of JEG-3 cells, which was associated with a downregulation of matrix metalloproteinases (MMP)-2/9. The in vivo findings provided further evidence of the inhibition of α-solanine on choriocarcinoma tumor growth. α-Solanine suppressed the xenograft tumor growth of JEG-3 cells, resulting in smaller tumor volumes and lower tumor weights. Apoptosis was promoted in xenograft tumors of α-solanine-treated mice. Moreover, α-solanine downregulated proliferative cellular nuclear antigen (PCNA) and Bcl-2 levels and promoted the expression of Bax. Collectively, α-solanine inhibits the growth, migration, and invasion of human JEG-3 choriocarcinoma cells, which may be associated with the induction of apoptosis.

## 1. Introduction

Human placenta choriocarcinoma is a highly malignant trophoblastic tumor characterized by abnormal trophoblastic hyperplasia, the absence of chorionic villi, hemorrhage, necrosis, and high metastatic potential [1,2]. Choriocarcinoma is a very rare neoplasm. The reported incidence of choriocarcinoma differs significantly in various regions; 202 instances per 100,000 pregnancies occur in China, and only two cases per 100,000 pregnancies in the United States [3]. Patients with choriocarcinoma often suffer from intrauterine growth restriction, preeclampsia, and even miscarriage [4]. Chemotherapy is commonly used to cure this disease, but there are risks, including the occurrence of secondary malignancies, nausea/vomiting, alopecia (hair loss), diarrhea, fevers, infections, and the need for blood transfusions [3,5]. Therefore, it is necessary to identify novel chemotherapeutic agents that have high efficacy and low toxicity for the successful management of human choriocarcinomas.

α-Solanine, rich in potato tubers and the nightshade plant, is a naturally occurring steroidal glycoalkaloid. Glycoalkaloids from plants have been found to defend against insects or herbivores [6]. The intake of high doses of α-solanine causes vomiting, diarrhea, and cardiac dysrhythmia in humans [7]. Recently, several studies have been reported that α-solanine exerts anti-cancer properties by inducing apoptosis and inhibiting cell growth, migration, and invasion in multiple cancer cells in vitro [8]. Apoptosis, or programmed cell death, is used remove aging and dead cells to maintain and stabilize homeostasis [9,10,11]. Bcl-2 and Bax are two important regulators in the process of cell apoptosis. Bcl-2 binds to Bax to form isomeric dimers that regulate the intracellular concentration of calcium (Ca^2+^) to exert anti-apoptotic effects [12]. The downregulation of Bcl-2 can induce apoptosis [13]. In addition, α-solanine has potential chemoprotective and chemotherapeutic effects on breast cancer in mice [14,15]. However, its effect on human choriocarcinoma is still unclear.

Our resent findings illustrated the toxic effects of α-solanine on human placental trophoblasts [16]. Combined with its anti-cancer properties, we hypothesized that α-solanine has a potentially inhibitory effect on the growth of human choriocarcinoma. Therefore, in the present research, we studied the effects of α-solanine on the cell growth, invasion, and migration of human choriocarcinoma JEG-3 cells in vitro. The anti-choriocarcinoma effect of α-solanine was verified using JEG-3 xenograft tumors in athymic nude mice. The effects of α-solanine on tumor growth and apoptosis were explored in vivo. This study demonstrates an inhibitory effect of α-solanine on the growth of human choriocarcinoma for the first time.

## 2. Results

### 2.1. α-Solanine Reduced the Cell Viability of JEG-3 Cells

**Figure 1:**
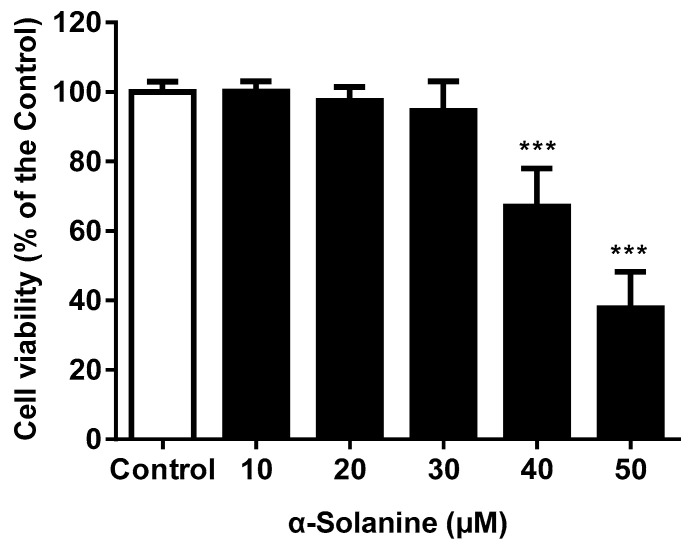
Effect of α-solanine on the viability of JEG-3 cells as analyzed by MTS assay. Data are represented as mean ± standard deviation (S.D.) from three independent experiments. Asterisks indicate significant differences compared with the control [0.5% *v/v* dimethyl sulfoxide (DMSO)] (*** *p* < 0.001).

As shown in Figure 1, the MTS [3-(4,5-dimethylthiazol-2-yl)-5-(3-carboxymethoxyphenyl)-2-(4-sulfophenyl)-2H-tetrazolium] assay showed that α-solanine decreased the cell viability of JEG-3 cells in a dose-dependent manner. At a concentration of 40 μM, α-solanine decreased cell viability to 67% of the control group. JEG-3 cell viability was not affected after 24 h of exposure to α-solanine in the concentration range tested (up to 30 μM). The expression of PCNA, a proliferation marker, was not significantly altered after α-solanine treatment at concentration of 30 μM (data not shown).

### 2.2. α-Solanine Suppressed the Colony Formation of JEG-3 Cells

**Figure 2:**
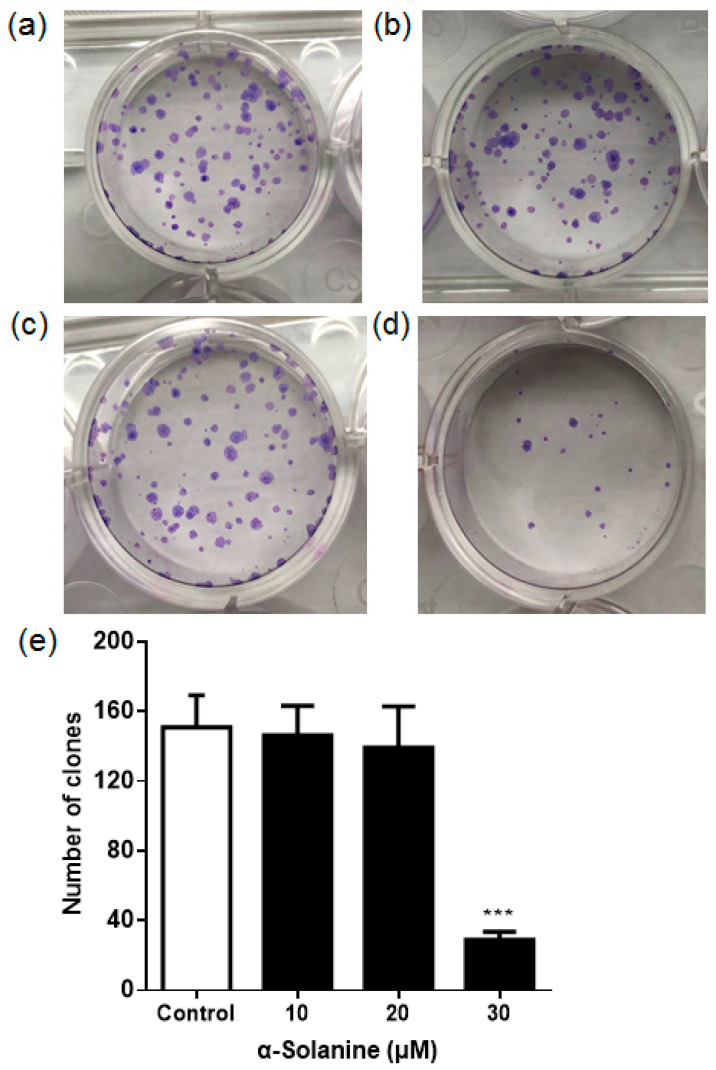
α-Solanine suppressed the colony formation of JEG-3 cells. Cell clones were stained with Giemsa stain solution. (**a**) Control group exposed to vehicle (0.5% *v/v* DMSO); (**b**) 10 µM α-solanine-treated group; (**c**) 20 µM α-solanine-treated group; (**d**) 30 µM α-solanine-treated group; (**e**) statistical analysis of the number of colonies. Data represent mean ± S.D. from three different experiments. Asterisks indicate significant differences between the α-solanine-treated group and the control (*** *p* < 0.001).

To evaluate the sensitivity of JEG-3 cells to α-solanine, cell survival rate was quantified by a colony survival assay. As shown in Figure 2, compared to the control group, the colony formation of JEG-3 cells was not affected in response to 10 µM or 20 µM α-solanine. However, the number of colonies was significantly decreased with 30 µM α-solanine treatment, which demonstrated that 30 µM α-solanine reduced the survival of JEG-3 cells.

### 2.3. α-Solanine Induced Apoptosis in JEG-3 Cells

**Figure 3:**
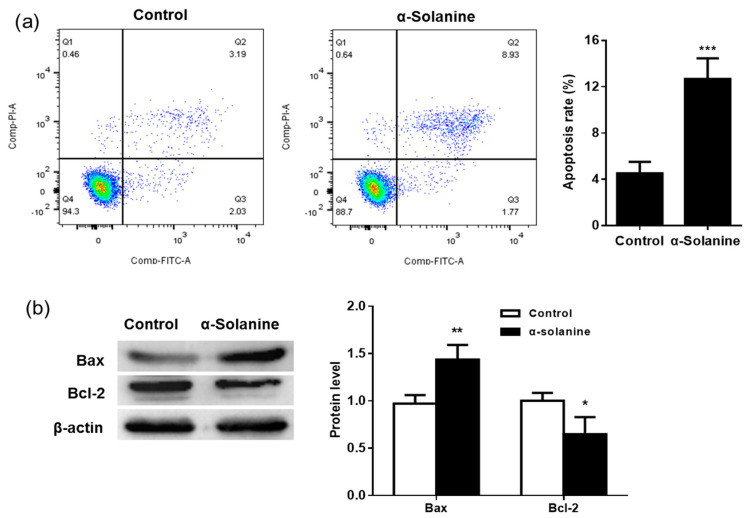
Effects of α-solanine on apoptosis in JEG-3 cells. JEG-3 cells were treated with 30 µM α-solanine for 24 h. (**a**) Cells were stained using Annexin V- fluorescein isothiocyanate (FITC) and propidium iodide (PI) and analyzed by flow cytometry. (**b**) The expression of the apoptotic proteins Bax and Bcl-2 were detected by Western blot. Data are expressed as the mean ± S.D. from three independent experiments. Asterisks indicate significant differences between the α-solanine-treated group and the control (0.5% *v/v* DMSO). * *p* < 0.05, ** *p* < 0.01, and *** *p* < 0.001.

Flow cytometry was used to determine apoptosis in JEG-3 cells. As shown in Figure 3a, α-solanine significantly increased the apoptotic rate of JEG-3 cells (*p* < 0.001), with a 2.8-fold increase when compared to the control group. Apoptotic cells are those positively stained by Annexin V-FITC as in quadrant (Q)2 (Annexin V-FITC+/PI+) and Q3 (Annexin V-FITC+/PI-). Moreover, the pro-apoptotic protein Bax was upregulated, while the expression of the anti-apoptotic Bcl-2 protein was decreased (Figure 3b).

### 2.4. α-Solanine Inhibited the Migration and Invasion of JEG-3 Cells and Downregulated the Expression of MMP-2/9

**Figure 4:**
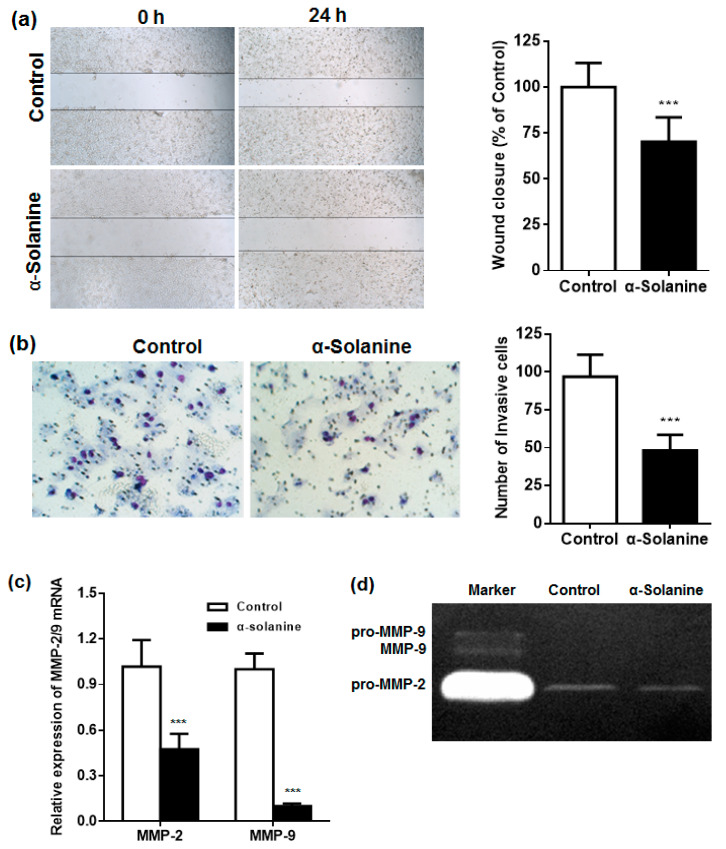
Effect of α-solanine on the cell migration and invasion abilities of JEG-3 cells. (**a**) Representative images of JEG-3 cells migrating into the wounded zone in the presence of 30 µM α-solanine or vehicle (magnification: 10×), and wound closure quantified in each group. (**b**) Representative images of JEG-3 cells invading through the filter in the presence of 30 µM α-solanine or vehicle (magnification: 100×), and quantitative analysis of the number of invasive JEG-3 cells. (**c**) The expression of matrix metalloprotein (MMP)-2/9 mRNA in 30 µM α-solanine or vehicle-treated JEG-3 cells. (**d**) Zymography of the cell culture medium of JEG-3 cells. All data are presented as mean ± S.D. (*n* = 3). *** *p* < 0.001 compared with the control.

A wound healing assay was performed to evaluate the potential effect of α-solanine on the migration of JEG-3 cells. The results show that α-solanine suppressed the migratory ability of JEG-3 cells into the scratched zone (*p* < 0.001) (Figure 4a). We observed a similar inhibitory effect of α-solanine on the invasive ability of JEG-3 cells. As shown in Figure 4b, only 49% of JEG-3 cells invaded across the Matrigel-coated filter when compared to that of the control. MMPs are important in extra-cellular matrix (ECM) degradation and the invasiveness of various cancer cells. The expression and activation status of MMP-2/9 were studied by RT–qPCR and zymographic analysis, respectively. As shown in Figure 4c, the expression of MMP-2/9 mRNA was significantly reduced in α-solanine-treated cells. Only pro-MMP-2 (inactive MMP-2) was detected in the culture medium of JEG-3 cells, which was significantly decreased in response to α-solanine treatment (Figure 4d). The active form MMP-2, pro-MMP-9 and MMP-9, were not detected in the culture medium.

### 2.5. α-Solanine Inhibited JEG-3 Cell Proliferation In Vivo

**Figure 5:**
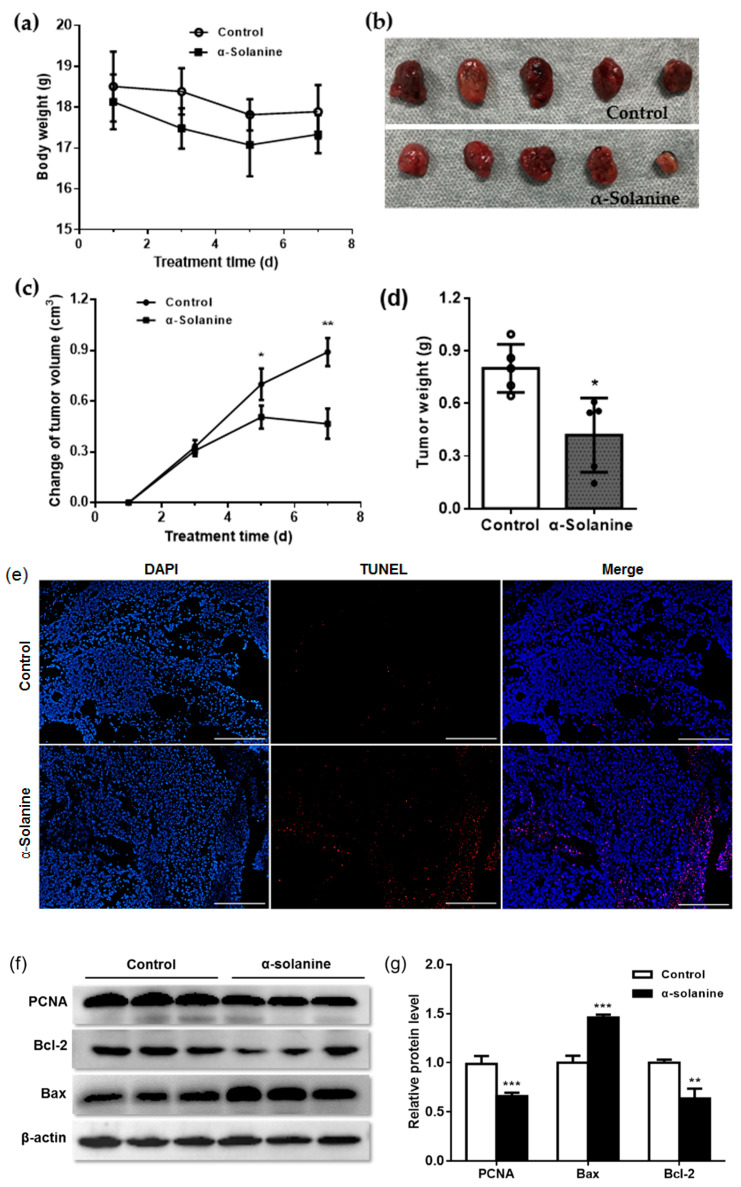
α-Solanine inhibited the growth of xenografted choriocarcinoma tumors in vivo. (**a**) Mouse body weight. (**b**) Xenograft tumors collected on day 7 post α-solanine treatment. (**c**) Changes in tumor volume. (**d**) Tumor weight. (**e**) Apoptosis as detected by TUNEL assay. (**f**,**g**) The expression of proliferating cell nuclear antigen (PCNA), Bcl-2 and Bax as detected by Western blot. Data are presented as mean ± S.D. Asterisks indicate significant differences between the α-Solanine-treated group and the control group. DAPI, 5. 4′,6-diamidino-2-phenylindole, * *p* < 0.05, ** *p* < 0.01, and *** *p* < 0.001.

The effects of α-solanine on the growth and apoptosis of JEG-3 cells were further investigated in vivo. We established a xenograft model by injecting JEG-3 cells into the right frank of athymic mice. Body weights showed no statistical difference between α-solanine-treated mice and control mice (Figure 5a). Xenograft tumor volume decreased on day 5 and day 7 post α-solanine treatment compared to the DMSO-treated group (*p* < 0.05) (Figure 5b,c). The weight of the xenograft tumors in the α-solanine group significantly decreased compared to that of the control group (*p* < 0.05) (Figure 5d). These results indicate that α-solanine inhibits xenograft growth of choriocarcinoma cells in nude mice. The terminal dUTP nick-end labeling (TUNEL) assay showed that the number of TUNEL-positive cells was significantly higher in α-solanine-treated tumors than in vehicle-treated tumors (Figure 5e). We further examined the expression of PCNA and apoptotic proteins in the xenograft tumors. The results showed that PCNA level (*p* < 0.001) and the expression of Bcl-2 protein (*p* < 0.001) decreased, while Bax level (*p* < 0.001) was upregulated in α-solanine-treated xenograft tumors compared with the vehicle-treated group (Figure 5f,g).

## 3. Discussion

Studies have shown that α-solanine exhibits toxicity in reproduction, causing abnormal physiological functions in the testes [17], oocyte maturation [18], and embryo and fetus development [19] in mammals. In addition to its toxic effects, recent studies have demonstrated that α-solanine acts as a potential compound for therapeutic treatment against various cancer cells [20,21].

Malignant proliferation is a biological characteristic of cancer cells. α-Solanine exerts anti-carcinogenic potential through inhibiting cell growth and inducing apoptosis in several cancer cells [22,23,24]. Here, we demonstrated that treatment of cultured choriocarcinoma cells with α-solanine suppressed cell proliferation in a dose-dependent manner, and α-solanine at a concentration of 30 µM or below did not significantly affect cell viability. However, 30 µM α-solanine inhibited colony formation in JEG-3 cells. In the colony formation assay, the number of cells was small (only 200 cells per well), which may increase the sensitivity of cells to α-solanine. Moreover, an animal model was established using malignant trophoblast JEG-3 cells grown in nude mice, in which fast-growing tumors developed in a short time [25,26]. The growth of the choriocarcinoma xenografts was significantly inhibited by α-solanine. PCNA is a marker of proliferation given its role in the replication of eukaryotic genomes [27]. The expression of the PCNA protein decreased in response to treatment with α-solanine in xenografts of JEG-3 cells in this study.

Apoptosis is a potential host defense mechanism against tumor cells. α-Solanine induces cell apoptosis and exhibits anti-tumor properties [28]. Our results showed that α-solanine promoted apoptosis in JEG-3 cells in vitro and altered the balance between Bcl-2 and Bax in choriocarcinoma xenografts in vivo, which may be involved in its anti-choriocarcinoma potential. Similar results have been reported in human colorectal cancer cells [28].

Tumor cell proliferation, migration, and invasion are closely associated with cancer metastasis [29,30]. The migration and invasion of cancer cells are often evaluated by wound-healing assays and Transwell assays, respectively [31]. α-Solanine can inhibit cell migration and invasion in several human cancers [32]. In human prostate cancer cells, α-solanine at non-toxic doses can markedly suppress cell invasion [33]. Here, we firstly demonstrated the inhibitory effect of α-solanine on migration and invasion in human choriocarcinoma cells. Proteolytic enzymes, like MMPs, are closely associated with disease progression and metastasis due to the degradation of the ECM in many cancers [34,35]. The inhibition of MMP-2/9 reduces tumor invasion and metastasis in cervical cancer cells [36,37], pancreatic cancer [38], and choriocarcinoma [26]. The levels of MMP-2/9 mRNA, as well inactive MMP-2 (pro-MMP-2), were decreased in α-solanine-treated JEG-3 cells, indicating that the inhibitory effect of α-solanine on the metastasis of choriocarcinoma cells may be associated with MMP-2/9. However, the active forms of MMP-2/9 were not detected in the cell culture medium. Similar results were reported in JEG-3 cells [39] and in triple negative breast cancer cells [40]. The role(s) of α-solanine in the activation of MMPs needs to be identified in future research.

Collectively, the current study reveals the inhibitory effect of α-solanine on the growth of human choriocarcinoma in vitro and in vivo. In our previous study, we found that human trophoblast-derived cell line HTR-8/SVneo was more sensitive to α-solanine than choriocarcinoma JEG-3 cells [16]. As a potential chemoprotective or chemotherapeutic agent, more studies are required to fully elucidate the molecular mechanism underlying the role of α-solanine against choriocarcinoma.

## 4. Materials and Methods

### 4.1. Chemicals and Reagents

α-Solanine, DMSO, and gelatin were obtained from Sigma-Aldrich (St. Louis, MO, USA). The CellTiter 96Ò AQueous Non-Radioactive Cell Proliferation (MTS) assay kit was purchased from Promega Corporation (Madison, WI, USA). Dulbecco’s Modified Eagle’s Medium (DMEM)/F12 medium and fetal bovine serum (FBS) were from Gibco-Invitrogen (Carlsbad, CA, USA). Paraformaldehyde (PFA), Giemsa stain solution, and radioimmunoprecipitation assay (RIPA) lysis buffer were purchased from Solarbio Science and Technology Co., Ltd. (Beijing, China). The bicinchoninic acid (BCA) protein assay kit and penicillin–streptomycin solution were obtained from the Beyotime Institute of Biotechnology (Shanghai, China). Matrigel and the Annexin V-FITC Apoptosis Detection Kit I were obtained from BD Biosciences (San Jose, CA, USA). TRIzol (as a common representative) reagent and Pierce^TM^ Protease Inhibitor Mini Tablets were purchased from Thermo Fisher Scientific Inc. (Waltham, MA, USA). The HiScript^®^II Q RT SuperMix for qPCR (+gDNA wiper) kit and Synergy Brands (SYBR) Green PCR Mastermix were obtained from Vazyme Biotech (Nanjing, China). Antibodies were obtained from the following: PCNA (Abcam, Cambridge, MA, USA), Bax and Bcl-2 (Proteintech, Chicago, IL, USA), horseradish peroxidase (HRP)-β-actin (Aksomics, Shanghai, China), and HRP-linked secondary antibody (Santa Cruz Biotechnology, Dallas, TX, USA). Enhanced chemiluminescence (ECL) kit (NcmECL Ultra kit) was purchased from New Cell and Molecular Biotech Co., Ltd. (Suzhou, China).

### 4.2. Cell Culture and Proliferation Assay

The culture of JEG-3 cells (a human choriocarcinoma cell line) has been described previously [26]. To study the cytotoxic effect of α-solanine on JEG-3 cells, cells were cultured in 96-well plates overnight, followed by treatment with different concentrations of α-solanine (10, 20, 30, 40 and 50 μM) or vehicle (0.5% *v/v* DMSO) for 24 h. Then, MTS reagent was added (20 µL each well) and incubated at 37 °C for 3 h. The absorbance at 490 nm was measured using a microplate reader (NanoQuant Infinite M200 Pro, Tecan, Mannedorf, Switzerland). The concentration and timepoint used in this study were determined in our previous study [16]. α-Solanine was dissolved in DMSO to prepare the stock solution and diluted to the indicated concentrations using cell culture medium. The maximal non-cytotoxic dose determined in this experiment was used in further research.

### 4.3. Colony Formation Assay

JEG-3 cells were cultured in 12-well plates (200 cells per well) overnight, then treated with various concentrations of α-solanine (10, 20 and 30 μM) or vehicle for 24 h. After treatment, cells were cultured in complete medium for 7 days, with media change every 2 days. Cell colonies were fixed with 4% PFA and stained with Giemsa stain solution. The number of colonies was counted.

### 4.4. Annexin V and PI Staining

The Annexin V-FITC Apoptosis Detection Kit I was used to analyze the apoptosis of JEG-3 cells according to the instructions from the manufacture. After 24 h of α-solanine treatment, cells were harvested, washed with phosphate buffered saline (PBS) and resuspended in 100 µL 1 × binding buffer containing 0.5 µg/mL Annexin V-FITC for 20 min. Subsequently, 200 µL 1 × binding buffer with 50 µg/mL propidium iodide (PI) was added to the cells for 5 min at room temperature in the dark. The analysis of cell apoptosis was performed on a FACSCalibur flow cytometer (BD Biosciences, Mountain View, CA, USA).

### 4.5. Western Blot

Total protein extracts from cells or tissues were prepared using RIPA buffer with the addition of a protease inhibitor cocktail. Protein concentration was determined by BCA protein assay. Equal amounts of protein were loaded and separated by SDS-PAGE, transferred onto a polyvinylidene difluoride membrane (Millipore, Bedford, MA, USA), blocked with 0.2% gelatin solution, and incubated with primary antibodies, including PCNA (1:1000 dilution), Bax (1:1000 dilution), Bcl-2 (1:1000 dilution), and HRP-β-actin (1:10,000 dilution) at 4 °C overnight. The membranes were washed 3 times in tris buffered saline with tween 20 (TBST), then incubated with a HRP-linked secondary antibody for 1 h at room temperature. The blots were developed using an ECL Ultra kit and visualized with the ChemiDoc XRS+ system (Bio-Rad, Hercules, CA, USA).

### 4.6. Wound Healing Assay

A wound healing assay was performed to assess cellular migratory ability as described previously [26]. Briefly, JEG-3 cells were seeded in 6-well plates. The cell monolayer was scratched using a 200 µL pipette tip and washed with serum-free medium. Then, cells were treated with 30 μM α-solanine or vehicle for 24 h. The wound region was photographed using a microscope. Wound closure was quantified using ImageJ software (National Institutes of Health, Bethesda, MD, USA).

### 4.7. Transwell Invasion Assay

A Matrigel invasion assay was performed to evaluate the invasive ability of JEG-3 cells as described previously with minor modifications [26]. JEG-3 cells in serum-free medium with 30 μM α-solanine or vehicle were seeded in the upper surface of Matrigel-coated Transwell inserts. Complete growth medium was added to the 24-well plates. After incubation for 24 h, the cells on the upper surface of the filter were removed, and those attached to the other side were fixed with methanol and stained using Giemsa staining solution. Cells were counted from five random microscopic fields using the ImageJ software.

### 4.8. Reverse Transcription and Quantitative RT-PCR

Total RNA was extracted using chloroform solution containing 20% TRIzol, and the RNA concentration was determined using a spectrophotometer (NanoDrop 2000, Thermo Fisher Scientific). cDNA was generated using the HiScript^®^II Q RT SuperMix for qPCR (+gDNA wiper) kit according to instructions from the manufacturer. qPCR was performed using 2 × SYBR Green PCR Mastermix in a Stepone Real-Time PCR system (Applied Biosystems, Foster City, CA, USA). The expression of the target genes was calculated using the 2^−ΔΔCT^ method and normalized to β-actin levels. Primer sequences were as follows: β-actin, forward 5′-CATCCGTAAAGACCTCTATGCCAAC-3′ and reverse 5′-ATGGAGCCACCGATCCACA-3′; MMP-2, forward 5′-CCCCGATGCTGATACTGA-3′ and reverse 5′-TGTCCGCCAAATAAACC-3′; MMP-9, forward 5′-AGCCAACTATGACCAGGAT-3′ and reverse 5′-TGCCACCAGGAACAGG-3′.

### 4.9. Zymography

Gelatinase activities of MMP-2/9 were assessed in the culture media as described previously [41]. The supernatants were harvested, and total protein concentration was measured by BCA protein assay. Samples were mixed with non-reducing SDS-PAGE loading buffer and run on a 10% SDS-PAGE gel. After electrophoresis, the gels were washed with TBST followed by a 1 h incubation with renaturing buffer (50 mM Tris (pH 7.5) and 2.5% Triton X-100) at room temperature. Then, gels were incubated in developing buffer (50 mM Tris (pH 8.0), 10 mM CaCl_2_, 0.2 M NaCl, 1 μΜ ZnCl_2_ and 1% Triton X-100) at 37 °C for 24 h. The gels were subsequently stained with Coomassie brilliant blue R-250 solution for 2 h and destained in a solution containing 50% methanol and 10% glacial acetic acid. The band densities were qualitatively analyzed using the ChemiDoc XRS+ system (Bio-Rad).

### 4.10. Animal Experiments

#### 4.10.1. Animal Treatment

Female athymic nude mice (BALB/c strain, 7 weeks old) were obtained from the Hunan SJA Laboratory Animal Corporation (Changsha, China) and housed under standard laboratory conditions with a 12 h light on/off cycle. All animal procedures were performed with the approval of the Ethical Committee of Animal Experiments, Hunan Agricultural University (No. 43201805, approved on 8 May 2018). No animals were subjected to unnecessary suffering in this study. The in vivo xenograft tumors were produced as described previously [26]. JEG-3 cells at a concentration of 5 × 10^6^ cells in 100 μL PBS were subcutaneously injected into the right flanks of mice. The mice were randomly divided into two groups (five mice per group) after the xenograft tumor formed on day 7 post JEG-3 cell injection. One group received α-solanine (5 mg/kg/day body weight) by intraperitoneal injection continuously for 7 d. The α-solanine dose was the same as that used in a recent study [28]. The other group received the same volume of vehicle (5% *v/v* DMSO). The first day of α-solanine treatment was designated as day 1. Tumor volumes were measured every other day by the measuring length (L) and width (W), and the size of the tumor volume was calculated as 0.5 × L × W^2^ [42]. Changes in tumor volume were analyzed in the two groups.

#### 4.10.2. Animal Sacrifice and Tissue Sampling

After treatment, animals were euthanized with a 2% isoflurane–98% air mixture and then sacrificed under anesthesia by cervical dislocation. The xenograft tumors were harvested, rinsed with ice-cold PBS, weighed, and divided into two pieces. One piece was frozen at −80 °C for Western blot analysis. The other piece was fixed in 4% PFA solution, embedded in paraffin wax, and sectioned (5 µm-thick) for the TUNEL assay.

#### 4.10.3. TUNEL Assay

The TUNEL assay was performed to detect in situ apoptosis in the tumor xenograft using a DNA Fragmentation Detection Kit (Beyotime) according to the instructions from the manufacturer. The dewaxed sections were incubated with DNase-free protease K (20 μg/mL, Beyotime) in 10 mM Tris (pH 7.5) at 37 °C for 20 min and washed with PBS. Then, sections were stained with the TUNEL staining mixture at 37 °C for 1 h in the dark, followed by a PBS wash and counterstaining with 4′,6-diamidino-2-phenylindole (DAPI). The stained sections were observed under a fluorescence microscope. Nuclei were stained blue, and apoptotic cells were stained red.

### 4.11. Statistical Analysis

Data are presented as the mean ± standard deviation (S.D.) from at least three independent experiments. The statistical analyses were performed by Student’s *t* test using GraphPad Prism software (version 5.0, San Diego, CA, USA).

## Data Availability

Data are available upon request, please contact the contributing authors.
